# Short-course radiation followed by mFOLFOX-6 plus avelumab for locally-advanced rectal adenocarcinoma

**DOI:** 10.1186/s12885-020-07333-y

**Published:** 2020-09-01

**Authors:** Ali Shamseddine, Youssef H. Zeidan, Malek Kreidieh, Ibrahim Khalifeh, Rim Turfa, Joseph Kattan, Deborah Mukherji, Sally Temraz, Kholoud Alqasem, Rula Amarin, Tala Al Awabdeh, Samer Deeba, Faek Jamali, Issa Mohamad, Mousa Elkhaldi, Faiez Daoud, Mahmoud Al Masri, Ali Dabous, Ahmad Hushki, Omar Jaber, Clement Khoury, Ziad El Husseini, Maya Charafeddine, Monita Al Darazi, Fady Geara

**Affiliations:** 1 Department of Internal Medicine, Division of Hematology/Oncology, Naef K. Basile Cancer Institute- NKBCI, American University of Beirut Medical Center, Beirut, Lebanon; 2 Department of Radiation Oncology, American University of Beirut Medical Center, Beirut, Lebanon; 3 Department of pathology and laboratory medicine, American University of Beirut Medical Center, Beirut, Lebanon; 4 Department of Medical Oncology, King Hussein Cancer Center, Amman, Jordan; 5 Department of Medical Oncology, Hôtel Dieu de France, Beirut, Lebanon; 6 Department of General Surgery, American University of Beirut Medical Center, Beirut, Lebanon; 7 Department of Surgical Oncology, King Hussein Cancer Center, Amman, Jordan; 8 Gastroenterology Department, King Hussein Cancer Center, Amman, Jordan; 9 Pathology Department, King Hussein Cancer Center, Amman, Jordan; 10 Department of Radiation Oncology, Hotel-Dieu de France Hospital, Beirut, Lebanon

**Keywords:** Rectal cancer, Radiotherapy, Chemotherapy, Immunotherapy, Neoadjuvant

## Abstract

**Background:**

Current standard practice for locally advanced rectal cancer (LARC) entails a multidisciplinary approach that includes preoperative chemoradiotherapy, followed by total mesorectal excision, and then adjuvant chemotherapy. The latter has been accompanied by low compliance rates and no survival benefit in phase III randomized trials, so the strategy of administering neoadjuvant, rather than adjuvant, chemotherapy has been adapted by many trials, with improvement in pathologic complete response. Induction chemotherapy with oxaliplatin has been shown to have increased efficacy in rectal cancer, while short-course radiation therapy with consolidation chemotherapy increased short-term overall survival rate and decreased toxicity levels, making it cheaper and more convenient than long-course radiation therapy.

This led to recognition of total neoadjuvant therapy as a valid treatment approach in many guidelines despite limited available survival data. With the upregulation (PDL-1) expression in rectal tumors after radiotherapy and the increased use of in malignant melanoma, the novel approach of combining immunotherapy with chemotherapy after radiation may have a role in further increasing pCR and improving overall outcomes in rectal cancer.

**Methods:**

The study is an open label single arm multi- center phase II trial. Forty-four recruited LARC patients will receive 5Gy x 5fractions of SCRT, followed by 6 cycles of mFOLFOX-6 plus avelumab, before TME is performed. The hypothesis is that the addition of avelumab to mFOLFOX-6, administered following SCRT, will improve pCR and overall outcomes. The primary outcome measure is the proportion of patients who achieve a pCR, defined as no viable tumor cells on the excised specimen. Secondary objectives are to evaluate 3-year progression-free survival, tumor response to treatment (tumor regression grades 0 & 1), density of tumor-infiltrating lymphocytes, correlation of baseline Immunoscore with pCR rates and changes in PD-L1 expression.

**Discussion:**

Recent studies show an increase in PD-L1 expression and density of CD8+ TILs after CRT in rectal cancer patients, implying a potential role for combinatory strategies using PD-L1- and programmed-death- 1 inhibiting drugs. We aim through this study to evaluate pCR following SCRT, followed by mFOLFOX-6 with avelumab, and then TME procedure in patients with LARC.

**Trial registration:**

Trial Registration Number and Date of Registration: ClinicalTrials.gov NCT03503630, April 20, 2018.

## Background

Rectal cancer continues to take a high toll in morbidity and mortality worldwide [1, 2]. In 2017, an estimated 39,910 new rectal cancer cases were diagnosed in the US [3]. The incidence of rectal cancer in the European Union is between 15 and 25 cases in a population of 100,000, and around 33% of these end up with death each year [4]. Over the last 20 years, stage II and III rectal cancers have shown a relatively steady 5-year OS of approximately 65% [5]. Despite the fact that the widespread use of screening has resulted in earlier identification and treatment of premalignant lesions and a decrease in incidence of rectal cancer [6], the SEER data estimated that by 2030 the colorectal incidence rate for the age group between 20 and 34 years will increase by 124.2% based on the previous colorectal data of cases under 50 years of age for the period between 1974 to 2010 of colorectal cancer [7].

Current standard of care for LARCs supports the results of the 5-year German (CAO/ARO/AIO) 94 trial and entails a multidisciplinary approach, where oxaliplatin was added to the preoperative CRT and adjuvant chemotherapy regimen, even though the concurrent use of oxaliplatin with chemoradiation is not the standard practice. This approach led to induce tumor regression, to increase the surgical negative margins (R0), and to decrease the local recurrence risk rate [8]. However, the EORTC 22921 trial follow up results showed no significant effect of adjuvant chemotherapy on both DFS and OS rates, adding the low compliance rate to chemotherapy with 82% pre-operatively and just 42.9% post-operatively [9, 10].

As a result of the low compliance and high complication rates observed with adjuvant chemotherapy, recent shifts toward a total neoadjuvant approach, where neoadjuvant instead of adjuvant chemotherapy is administered in an attempt to increase the pCR rate, which is of major prognostic impact in rectal cancer [11].

The recent concept of TNT in locally advanced rectal cancer was first tested in a clinical trial by Chau et al. which showed an 88% objective tumor control rate with neoadjuvant capecitabine/oxaliplatin [12, 13]. Recent trials have also explored this concept with results revealing promising pCR rates using TNT. Garcia-Aguilar et al. concluded in a phase II trial that patients who received 6 cycles of mFOLFOX6 between CRT and TME procedure resulted in an increase in pCR, reaching 38%, with better compliance to chemotherapy and without a significant increase in surgical complications compared to other groups who received less or no chemotherapy cycles in between [14]. Cercek et al. compared 320 patients receiving regular CRT to 308 patients receiving TNT, the CR rate was 36% in the latter group as compared to 21% in the first one [15]. Moreover, Bahadoer et al. showed in the randomized RAPIDO trial that the TNT regimen is superior to the standard preoperative CRT and surgery in pCR rates [16]. Furthermore, Garcia-Aguilar et al. showed in the ORPA trial that the TNT regimen can form the basis of organ preservation in rectal cancer management [17]. These substantial rates of tumor regression and pCR suggest that it’s more favorable to use of this treatment modality i.e., Chemotherapy and CRT before planned surgery of LARC. This could be secondary to the better compliance to chemotherapy experienced with TNT and to the > 8 weeks-delay between radiotheraoy and surgery, that increased the odds of pCR [18]. Findings of these studies provided additional support for the NCCN guidelines that classify TNT as an acceptable treatment modality for rectal cancer [19].

Although preoperative CRT and neoadjuvant chemotherapy followed by TME seems promising in locally advanced rectal cancer patients, the superiority in OS of short course radiation followed by neoadjuvant chemotherapy over preoperative CRT was not maintained after 8 years [20]. outcomes remain poor with a 3-year DFS of approximately 50%. Also, although this strategy greatly decreases the risk of loco-regional recurrence, the fact that late development of distant metastatic tumor spread is still common, requires innovative systemic strategies to overcome disease progression. For instance, while local recurrence rates for locally advanced rectal cancers were stable at 5 to 6% following the combination strategy used, distant recurrence rates were found to be around 25%, and metastases were considered the main cause of death [21, 22].

Conventionally, oxaliplatin was known to induce DNA damage and crosslinks ultimately leading to apoptosis. Furthermore, in mice, oxaliplatin was found to stimulate an increase in cytotoxic T cells and support an anti-tumor immune response. The immunogenic cell death is the interaction between chemotherapy and immune response, where oxaliplatin induces a change in the tumor microenvironment through activating cytotoxic T cells and cancer cells expression of MHC class I and immune checkpoint molecules. This leads to the recognition of cancer cells by the immune response [23, 24]. Moreover, adding 5-FU to oxaliplatin augment anticancer immune response through deactivating the myeloid derived suppressor cells that in turn downregulate tumor cell growth and angiogenesis [25].

In a multicenter phase II trial, Marco et al. demonstrated that delaying the chemoradiation-to-surgery period and increasing the number of neoadjuvant chemotherapy cycles caused an increase in the pCR rates [26]. Furthermore, a recent phase III randomized trial explored the effect of delaying surgery beyond 8 weeks from finishing radiation on pCR rates in LARC patients. A significant increase in the pCR rate with delayed TME (> 8 weeks from RT) was noted, reaching 28.6%, in comparison to 10% in the other group (< 8 weeks from RT) [27]. Finally, in the Stockholm III trial, short-course radiotherapy with delayed surgery resulted in no significant difference in locoregional disease control, pCR rates, distant recurrence and OS in comparison to long-course radiotherapy [28]. This advocates the use of SCRT and to benefit from the abscopal effect. The abscopal effect is the elimination of distant metastatic tumor cells by an immune response triggered by targeting the tumor with radiation locally. It was hypothesized that the destruction of tumor cells by radiation causes the release of tumor antigens that induce an immune response locally and distally [29]. Moreover, a correlation was noted between radiation dose per fraction and the strength of the abscopal effect. In mice, Camphausen et al. showed that 50 Gy in 5 fractions had a stronger abscopal effect than 24 Gy in 12 fractions [30]. In another study, 16 Gy in 2 fractions caused a higher increase in interferon levels and PD-L1 expression in comparison to 20 Gy in 10 fractions. This advocates for the use of high dose per fraction (hypofractionated) radiation with immune checkpoint inhibitors [31].

In an attempt to further increase the pCR and improve outcomes in rectal cancer patients, we proposed to investigate a novel approach of combining immune checkpoint inhibitors with systemic chemotherapy after SCRT. Baeten et al. showed that short course radiotherapy was significantly superior to long course chemoradiation in increasing cytotoxic T cells in the tumor biopsy post treatment in patients with rectal cancer [32]. Furthermore, chemoradiation was found to increase PD-L1 expression, in the tumor and its invasive margin, in patients with rectal adenocarcinoma [33]. As a result of this increase in both, PD-L1 and its PD-1 receptor can be utilized as a potential therapeutic target of immunotherapy through proposing to use PD-1/PD-L1-inhibiting drugs.

Preliminary pharmacokinetic and clinical safety data from cohorts of phase I trials, the phase II pivotal trial (EMR100070–003), and the ongoing phase III trials support the efficacy of avelumab in combination with other treatment such as Axitinib in Renal Cell carcinoma (RCC). In addition, Avelumab is now approved in Merckel-Cell carcinoma (MCC) and studies are still ongoing in Lung cancer. Responses with such combinations were observed early during treatment and appeared to be durable in nature, lasting for > 1 year in several of the cohorts [34, 35]. This further supports the idea of combining PD-1/PD-L1 inhibitors with CRT for improved clinical efficacy in the management of rectal cancer patients.

Based on the above, we initiated this phase II clinical study to evaluate the pCR rate following 5 fractions of SCRT (for a total of 25 Gy), followed by 6 cycles of mFOLFOX-6 chemotherapy combined with avelumab immunotherapy (given every 2 weeks), and then TME procedure in patients with LARC.

## Methods/design

### Study design

**Fig. 1:**
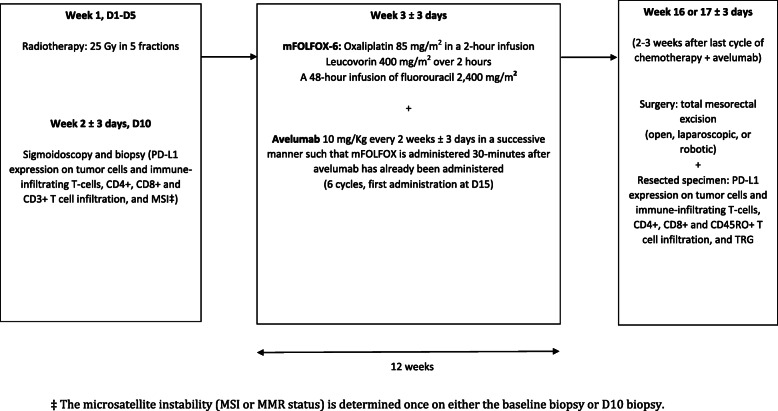
Study design

The study is an open-label, single-arm multicenter prospective stage-II phase-II trial investigating SCRT (25 Gy in 5 fractions), followed by 6 cycles of mFOLFOX-6 chemotherapy plus avelumab immunotherapy (10 mg/kg), and later by TME procedure (open, laparoscopic, or robotic) in patients with locally-advanced, potentially resectable rectal adenocarcinoma. The treatment algorithm is presented in Fig. 1.

### Study objectives

The primary outcome measure is the proportion of patients who achieve a pCR defined as no viable tumor cells on the resected specimen obtained during the TME procedure at week 16. Secondary objectives are to evaluate the PFS at 3 years, the tumor regression grade after cytotoxic treatment (i.e. tumor response to treatment), the extent of CD4+, CD8+, and CD3+ T-cell infiltration, and changes in PD-L1 expression on tumor cells and TILs. Furthermore, quality of life (QoL) and toxicity profile are secondary endpoints.

### Trial organization

This trial is principal-investigator initiated and sponsored by AUB. A total of three centers from two countries, Lebanon and Jordan, are involved in the study. In Lebanon, participating sites are the American University of Beirut Medical Center (AUBMC) and Hotel Dieu De France (HDF). In Jordan, the participating site is King Hussein Cancer Center (KHCC). Each center is expected to enroll 14–15 eligible patients for a total of 44 patients.

### Trial duration

The study duration is expected to be 4.5 years if the 2 stages of the study were completed. This includes 18 months of enrolment and 3 years of follow-up. The duration of this study depends on the results of the interim analysis, i.e. the probability of early termination, if only 2 or fewer patients achieves pCR at the end of stage one.

For each patient, the participation will last 36 months, with a treatment period extending for 16 weeks and a follow-up period after surgery extending for 3 years and occurring every 3 months. The first patient’s first visit has already occurred on 20 July 2018, and last patient last visit is expected to be on 02 Nov2023. Database lock and key statistics are expected to take place on 02 January 2024 and 02 March 2024, respectively. The clinical study report is expected to be finalized on 02 June 2024.

### Coordination and monitoring

The trial is coordinated by Phoenix Clinical Research, a contract research organization (CRO) responsible for overall trial management, database development, quality assurance, and trial registration at ClinicalTrials.gov (mFOLFOX-6). Designated principal investigators, co-principal investigators, and coordinators are assigned to each trial enrollment site.

A monitoring committee performs on-site monitoring of recruited patients in accordance with the international guidelines on Good Clinical Practice (ICH-GCP) and the revised version of the Declaration of Helsinki [36].

### Statistics

Assuming that the historical rate of pCR with SCRT followed by systemic 5FU-oxaliplatin chemotherapy is 16 to 19% [37] and that the rate of pCR with the addition of avelumab increases to 35%, Simon’s two-stage design [38] will be used. The null hypothesis that the true response rate is ≤16% will be tested against a one-sided alternative as follows:

- H_0_: p (pCR) ≤0.16
- H_1_: p (pCR) ≥0.35

The use of the Simon two-stage design enables an interim analysis for both efficacy and safety to be performed following treatment of the first 13 eligible patients. As such, an interim analysis is expected to take place at the end of stage one (around July 2019), after the 13th patient will have completed his/her TME procedure. If this analysis reveals 2 or fewer patients with a pCR, the study will be discontinued. Otherwise, 31 additional patients will be accrued and the sample size increased to 44 patients. This sample size ensures that we achieve a total of 36 patients who are eligible for the primary efficacy analysis. If at least 10 patients end up with a pCR, the null hypothesis will be rejected. This design yields a type 1 error rate of 0.05 and a power of 0.8 when the true pCR rate is 35%.

The primary endpoint will be presented by number and proportion of patients who achieve pCR along with the corresponding one-sided 95% confidence interval (Wilson Score Method). The median PFS at 3 years will be estimated using the Kaplan-Meier method, and it will be presented along with its 95% confidence interval. The remaining exploratory variables, including the extent of T-cell infiltration and PDL-1 expression on tumor cells and TILs, will be analyzed according to their scale of measurement by using mean ± standard deviation or frequency distribution for numeric and categorical variables, respectively. Frequency distribution for AEs and (serious adverse events) SAEs will be presented per cycle and per patient.

### Drug supply

The investigational medicinal product (IMP) will be shipped to the investigator/hospital pharmacist by Merck KGaA, Darmstadt, Germany in accordance with the local requirements and as soon as the initiation of the site is validated by the principal investigator. The shipment frequency to each site will be related to the consumption rate per site and will be adapted accordingly. Taking into consideration the expiry / re-test date of the IMP**.**

### Patient selection

In this study, a total of 44 patients with locally-advanced, potentially resectable rectal adenocarcinoma will be enrolled with the condition that they meet the study inclusion criteria and none of the exclusion one. Please refer to Table 1 for the inclusion and exclusion criteria.

**Table 1:** Inclusion and Exclusion Criteria

Inclusion Criteria	Exclusion Criteria
1) Signed informed consent form. 2) Patients aged ≥18 years. 3) Locally-advanced rectal cancer cT2 N1–3, cT3/T4a N0–3 4) < 12 cm from anal verge. 5) Histologically proven rectal adenocarcinoma. 6) ECOG performance score ≤ 1. 7) Have adequate organ function by meeting the following: • Absolute neutrophil count (ANC) ≥ 1.5 × 109/L; • Platelet count ≥100 × 109/L; • Hemoglobin ≥9 g/dL; • Total bilirubin level ≤ 1.5 × the upper limit of normal (ULN) range; • AST and ALT levels ≤2.5 × ULN or AST and ALT levels ≤5 x ULN (for subjects with documented metastatic disease to the liver); • Estimated creatinine clearance ≥30 mL/min according to the Cockcroft-Gault formula (or local institutional standard method). 8) Negative serum or urine pregnancy test at screening for women of childbearing potential.	1) Distant metastasis (M1). 2) Patients with T2 N0 or T4b. 3) Recurrent rectal cancer. 4) Symptoms or history of peripheral neuropathy. 5) Prior radiotherapy or chemotherapy. 6) Current use of immunosuppressive medication except for the following: - Intranasal, inhaled, topical steroids, or local steroid injection (e.g., intra-articular injection); - Systemic corticosteroids at physiologic doses ≤10 mg/day of prednisone or equivalent; - Steroids as premedication for hypersensitivity reactions (e.g., CT scan premedication). 7) Concurrent treatment with a non-permitted drug. 8) Active autoimmune disease that might deteriorate when receiving an immuno-stimulatory agent. 9) Vaccination within 4 weeks of the first dose of avelumab and while on trials is prohibited except for administration of inactivated vaccines. 10) Active infection requiring systemic therapy. 11) Known history of testing positive for the human immunodeficiency virus or known acquired immunodeficiency syndrome. 12) Hepatitis B virus (HBV) or hepatitis C virus (HCV) infection at screening (positive HBV surface antigen or HCV RNA if anti-HCV antibody screening test positive). 13) Known prior severe hypersensitivity to investigational product or any component in its formulations, including known severe hypersensitivity reactions to monoclonal antibodies (NCI CTCAE v4.03 Grade ≥ 3). 14) Clinically significant (i.e., active) cardiovascular disease: cerebral vascular accident/stroke (< 6 months prior to enrollment), myocardial infarction (< 6 months prior to enrollment), unstable angina, congestive heart failure (≥ New York Heart Association Classification Class II), or serious cardiac arrhythmia requiring medication. 15) Persisting toxicity related to prior therapy (NCI CTCAE v. 4.03 Grade > 1); however, alopecia, sensory neuropathy Grade ≤ 2, or other Grade ≤ 2 not constituting a safety risk based on investigator’s judgment are acceptable. 16) Prior organ transplantation including allogenic stem-cell transplantation. 17) Any psychiatric condition that would prohibit the understanding or rendering of informed consent.

^a^ The microsatellite instability (MSI or MMR status) is determined once on either the baseline biopsy or D10 biopsy

### Treatment plan

Patients who fulfil the eligibility criteria will be informed of all the details related to the study procedures. Only those who voluntarily accept to participate in the study and sign the informed consent will be enrolled. They will receive SCRT (a total of 25 Gy in 5 fractions), followed 2 weeks later by mFOLFOX-6 plus 10 mg/kg avelumab (6 cycles given at a rate of one cycle every 2 weeks, in a successive manner such that mFOLFOX is administered 30-min after avelumab has already been administered), then TME procedure (open, laparoscopic, or robotic) around week 16.

#### Visit 1

During this visit, the informed consent is obtained by the investigator or research fellow assigned to the site. The investigator/ research fellow obtains medical history from the patient and performs a complete physical examination, including a digital rectal exam. QoL assessment is performed by asking the patient to complete an Arabic or English version of the FACT-C questionnaire [39, 40]. Baseline laboratory tests including free T4, TSH, Hepatitis B virus surface antigen, and Hepatitis C virus antibodies are also ordered to ensure normal results prior to chemotherapy and immunotherapy administration. The patient will be asked to provide tissue blocks or at least 7 slides of the baseline biopsy specimen upon which diagnosis was based. These will be sent to AUBMC where the participating pathologist will proceed with the pathologic evaluation. PD-L1 expression on tumor cells and TILs will be assessed by the pathologist at AUBMC. Also, CD4+, CD8+ and CD3+ T cell infiltration will be quantified in mm^2^ in the most abundant tumor-infiltrating area in both, the stroma and the tumor, of the baseline biopsy. Microsatellite instability, MSI or MMR status, will be evaluated once on either the baseline biopsy or day 10 (D10) biopsy, and the predictive markers to be assessed are: MLH-1, MSH-2, MSH-6, and PMS-2.

#### Visits 2–6 (week 1 ± 3 days; day 1–5)

SCRT will be administered for 5 days from day 1 (D1) to D5 during week 1 (visits 2 to 6) by the participating radiation-oncologist in the corresponding site. Either 3D conformal or in intensity-modulated radiotherapy (IMRT) treatment planning may be used. Patients are to be placed in a position that best suits the technique used and ensures immobilization and displacement of normal tissues. The daily dose will be 5 Gy to a total dose of 25 Gy.

#### Visit 7 (week 2 ± 3 days; day 10)

Sigmoidoscopy will be performed and a biopsy taken by the participating gastroenterologist in the corresponding site. A PD-L1 expression on tumor cells and TILs will be assessed by the pathologist at AUBMC. Also, CD4+, CD8+ and CD3+ T cell infiltration will be quantified in mm^2^ in the most abundant tumor-infiltrating area in both, the stroma and the tumor, of the D10 biopsy. Microsatellite instability (MSI or MMR status) will be evaluated once on either the baseline biopsy or D10 biopsy, and the predictive markers to be assessed are: MLH-1, MSH-2, MSH-6, and PMS-2.

#### Visits 8–13 (week 3 ± 3 days to week 13 ± 3 days; day 15+)

mFOLFOX-6 chemotherapy plus avelumab will be administered every 2 weeks for 6 cycles. Pre-medications are administered first. Avelumab at a dose of 10 mg/kg is administered next, followed 30 min later by mFOLFOX as follows: 85 mg/m^2^ of oxaliplatin in a 2-h infusion, 400 mg/m^2^ of leucovorin over 2 h, followed by a 48-h infusion of fluorouracil 2400 mg/m^2^. Hematologic and biochemical laboratory tests are ordered prior to every cycle. During the visit, the investigator/ research fellow assigned at each site performs a complete physical examination and assesses QoL of participants by asking them to complete either an Arabic or English version of the FACT-C questionnaire [39, 40]. AEs that occur during or after the cycle administration are collected.

Visit 13 is the end of treatment visit which includes, in addition to the procedures mentioned above, an assessment of tumor markers (CEA and CA 19–9).

In case of premature discontinuation of the study treatment, a visit should be scheduled as soon as possible, but no later than 14 days from the last day of study medication, at which time all of the assessments listed for the end-of-treatment visit will be performed. An end-of-treatment eCRF page should be completed on-which the date and reason for stopping the study treatment should be provided.

#### Visit 14 (week 16 or 17 ± 3 days)

Three to four weeks after last cycle of mFOLFOX-6 plus avelumab, an open, laparoscopic, or robotic TME is performed at the corresponding site. An optional pelvic MRI might be ordered prior to surgery to evaluate the patient’s disease status. All TME procedures will be video recorded and the corresponding videotapes and images of the resected specimens are to be provided to AUBMC. All specimens are to be processed and graded using the recommendations of the College of American Pathologists [41, 42]. The excision specimen will be provided to the pathology department at AUBMC who will document, using the Becker et al. tumor regression grading (TRG) system, whether the patient achieved a pCR, defined as no viable tumor cells on the resected specimen [43]. PD-L1 expression on tumor cells and TILs will be assessed at AUBMC. Also, CD4+, CD8+ and CD3+ T-cell infiltration will be quantified in mm^2^ in the most abundant tumor-infiltrating area in both, the stroma and the tumor, of the tumor excision specimen. Frequency, grade, and attribution of surgical complications to the neoadjuvant treatment will be assessed using the Clavien-Dindo classification.

#### Follow-up visits

Physical examination, follow-up laboratory tests including tumor markers (CEA, Ca19–9), AE collection, QoL assessment using completed FACT-C questionnaire, and disease status evaluation are to be completed every 3 months for 3 years after the surgical procedure.

### Assessment of therapeutic efficacy

The primary efficacy endpoint is the proportion of patients who achieve a pCR defined as no viable tumor cells on the resected specimen. It will be assessed on all patients who received at least one IMP administration and who have undergone surgical resection. As mentioned before, in order to document whether a patient achieves a pCR, the Becker et al. tumor regression grading system will be used to categorize the amount of regressive changes after cytotoxic treatment and estimate the percentage of residual tumor in relation to the previous tumor site as follows [43]:

- 0. No residual tumor/ tumor bed + chemotherapy effect;
- 1. < 10% residual tumor/ tumor bed + chemotherapy effect;
- 2. 10–50% residual tumor/ tumor bed + chemotherapy effect;
- 3. > 50% residual tumor/ tumor bed **±** chemotherapy effect.

Evaluation of patients’ response to treatment is a secondary efficacy endpoint that will also be evaluated by obtaining the TRG just after surgery.

As for the other secondary efficacy endpoint, PFS at 3 years, it will be estimated using Kaplan Meier method for all patients with at least one IMP administration, whether they have undergone surgery or not.

## Discussion

Combination therapy with preoperative CRT, followed by TME procedure, and then adjuvant chemotherapy comprises the cornerstone of treatment in stages II and III rectal cancer. Although this has resulted in an improvement in tumor regression and a decreasein the risk of local recurrence [44], use of adjuvant chemotherapy has been accompanied by low compliance rates, manifested by patients not receiving or completing the planned treatment.

Many phase II and ongoing phase III trials adapted the strategy of administering neoadjuvant, rather than adjuvant, chemotherapy, and results have revealed marked improvement in pCR in TNT arms when compared to the regular CRT arms, reaching up to 38% in some cases [8, 14, 15, 21]. In addition to the results of the randomized RAPIDO trial that showed a doubling in the pCR rates with the TNT regimen in comparison to preoperative CRT [16]. This led to recognition of TNT as a valid treatment approach in rectal cancer by the NCCN [19].

Based on our experience with TNT at AUBMC, outcomes seem promising. Of the total 16 patients who underwent TNT since 2016, 10 have received 3–6 cycles of FOLFOX followed by long course CRT (Capecitabine + XRT 28 fractions), 3 have received 3–6 cycles of XELOX followed by long course CRT (Capecitabibne + XRT 28 fractions), 2 have received short course CRT followed by 6 cycles of FOLFOX, and 1 has received short course IMRT (5 fractions) followed by 2 cycles of XELOX. 44% of patients (7 of 16) had complete response (CR), 12% of patients (2 of 16) had down-staging from T3N1M0 to pT1-2 N0 and T4bN1M0 to pT1-2 N0, respectively, and 44% of patients (7 of 16) had incomplete response. In addition, only 25% of patients (4 of 16) had relapse later on.

Although TNT seems promising in LARC patients, the POLISH II trial after 8 years showed no superiority of TNT over CRT in survival and pCR rates. This may be due to the initial design of the study where patients only received 3 cycles of neoadjuvant chemotherapy [20]. Moreover, Fokas et al. showed in a randomized trial that sequence of TNT affects the clinical outcomes of patients, where CRT followed by neoadjuvant chemotherapy results in a higher pCR rate [45]. In an attempt to further increase the pCR and improve outcomes in rectal cancer patients, we proposed the use of a novel approach of combining immune checkpoint inhibitors with systemic chemotherapy after radiation which may overcome these limitations [46–49]. We initiated this phase II clinical study to evaluate the pCR rate following SCRT (5 fractions), followed by mFOLFOX-6 chemotherapy combined with 10 mg/kg of avelumab immunotherapy (6 cycles), and then a TME procedure in patients with LARC.

Innovative studies aimed at testing the efficacy of using anti PD-1/PD-L1 in combination with CRT in cancer patients showed promising result [46–49]. Indeed, PD-L1 expression and CD8+ TILs density significantly increased after neoadjuvant CRT in a matched comparison analysis of preoperative CRT-induced alterations on pre-CRT biopsy and post-CRT resected specimens of rectal cancer patients. It was also noted that patients whose PD-L1 expression was consistently high both, before and after CRT, experienced less elevation in CD8+ TILs compared to the rest of the groups [46–48]. These results suggest that CRT causes an up-regulation of anticancer immunity by increasing the CD8+ TIL density. They also support the idea that this increase in TIL density can be the reason behind the elevation in PD-L1 expression in tumor cells [50].

In the light of the marked increase in both after CRT, PD-L1 and its PD-1 receptor can be viewed as potential therapeutic targets, and through proposing using PD-1/PD-L1-inhibiting drugs. This is supposed to enhance the long-term antitumor effects of therapy in cancer patients [46–49], especially that the use of inhibitors of these cell surface markers in cholangiocarcinoma have played a potential therapeutic role in enhancing the immune killing of cancer cells by removing the inhibitory function of PD-1 on T cells [49]. Although previous trials involving the use of PD-1/PDL1 checkpoint inhibitors in colorectal cancer patients could not demonstrate a clear benefit [51], we rely on the fact that avelumab 10 mg/kg once every 2 weeks has demonstrated meaningful clinical activity across various treatment settings and tumor types, including MCC and RCC. For instance, preliminary pharmacokinetic and clinical safety data from cohorts of phase I trials, the phase II pivotal trial (EMR100070–003), and the ongoing phase III trials support the use of this dose of avelumab in combination with other treatment options, with responses being observed early during treatment and appearing to be durable in nature, lasting for > 1 year in several of the cohorts [34, 35]. As a result, a dose of 10 mg/kg IV once every 2 weeks was considered to have a favorable risk benefit profile for our present study.

With the promising results from trials that evaluate the combination of radiation therapy and immunotherapy in many cancer types, comes the question of the choice of when to start immunotherapy with respect to radiation therapy. Most reported cases of abscopal effects happened when radiation therapy was given either with or after cytotoxic T-lymphocyte–associated antigen 4 (CTLA-4) blockade [52–55]. Data from the ProHA-TRAMP model reveals that the up-regulation of anti-tumor effects was most optimal when a tumor vaccine was given within a narrow window of 3–5 weeks after radiation therapy [56, 57]. Of note, while radiation therapy-induced immunogenic cell death occurs within 1–3 days in breast tumor cells, anti-tumor immunity to melanoma is improved when anti-CTLA-4 antibody precedes radiation therapy [52, 58, 59]. In our study protocol, immunotherapy will be concomitantly administered with mFOLFOX6 2 weeks from the start of SCRT as we assume that this provides enough time for the expected upregulation in PDL-1 expression and TIL densities to occur and synergize with immunotherapy.

Previous studies stressed on the role of RT as an immune adjuvant [60–63]. For instance, CRT has been shown to increase the tumor antigen load, their related receptor molecules, and danger-related signal molecules as part of a long cascade of immune responses culminating in immunogenic cell death [64]. By increasing tumor cell lysis at the level of the localized treatment site, high doses of radiation result in the release of tumor-associated antigens (TAA) in the process. These antigens transported by antigen presenting cells (APCs) [65, 66], that once activated by pro-inflammatory cytokines, migrate to tumor-draining lymph nodes to activate cytotoxic T-lymphocytes (CTL) and mobilize them against tumor cells [67]. Indeed, abscopal effect is defined as the ability of radiation delivered to a local site to minimize or eradicate metastases at distant sites, outside the scope of the localized treatment [29]. This nonspecific eradication of distant tumors and metastases could be related to the increase in the levels of pro-inflammatory cytokines and chemokines released, the immune cells and tumor tissues in the system, following exposure to radiation [66].

Based on the results of several trials (Polish and Stockholm III) [20, 28], SCRT showed equivalent results to CRT for loco-regional control beside its potential immunogenicity. Also, current NCCN guidelines for the management of locally-advanced disease support the use of SCRT as a valid option, especially with data revealing tumor down-staging with the short-course [19, 68].

As for the timing of the TME procedure, Akgun et al. showed that randomized patients LARC localized within 12 cm of the anal verge into, an arm undergoing TME within 8 weeks and another arm undergoing the procedure after 8 weeks following CRT, showed improved stage regression and pCR when the interval between CRT and surgery exceeded 8 weeks [27]. Similarly, results from another study that randomized patients into two arms, with 6- and 12-week waiting periods, are available in an abstract form and show significantly higher pCR rates in the latter group [69]. Findings suggesting higher pCR and stage regression rates in long-interval groups are expected, especially that there is more time for the biological effects of radiation therapy to occur. For instance, despite the fact that DNA is damaged during irradiation, tumor cell lysis does not occur until weeks after irradiation [70–72]. By planning the TME procedure to be performed around 16 weeks after the start of radiation therapy, we give enough time for these effects to take place.

Our primary endpoint is to evaluate the pCR rate following pre-operative treatment, especially with recent data suggesting it as being a surrogate for DFS in locally-advanced rectal cancer [73]. This will enable us to test the hypothesis that the addition of avelumab to mFOLFOX-6 chemotherapy, administered following SCRT for locally-advanced rectal cancer, will improve the post-operative outcomes of the disease.

The management of rectal cancer continues to be challenging. Despite the plethora of evidence suggesting significant improvement in local recurrence rates in rectal cancer patients with advances in preoperative chemotherapy and surgery, distant metastases continue to represent a major problem. In fact, the combined statistical analysis data from five European randomized controlled trials showed that the 5-year distant metastasis rate was 30.8% in 2759 recruited patients [22]. We hope that with the regimen suggested in this protocol, a significant improvement in pCR and outcome will be obtained.

## Data Availability

Data that support the findings of this study are stored in a secured server, in a manner that the data is not publicly available but restricted to the acknowledged study personnel as per study protocol. Data processing, from data collection to database lock, will be carried out in accordance with Good Clinical Practice. The database structure, data entry manual, coding rules, and computerized validation are defined in a Data Management Plan. The database and data entry screens will be created in software specifically designed for clinical data management in compliance with ICH-E6 requirements. All eCRFs received in the Data Management Unit will be tracked by the Data Manager. The consistency of data will be checked by computerized programs and related queries will be generated for resolution by the Investigator. The database will then be updated accordingly. At the end database lock will be performed by the assigned data management team who will, consequently, be responsible for transferring the cleaned data into a statistical software for analysis. To access raw data, a request should be sent to the PI, Dr. Ali Shamseddine, via e-mail as04@aub.edu.lb, stating the reason for requesting the data and the list of variables needed. The PI will revise the request and grant permission accordingly. All data will be de-identified and encrypted.

## Abbreviations

CRT: Chemoradiotherapy
TME: Total mesorectal excision
pCR: Pathologic complete response
SCRT: Short-course radiation therapy
RT: Radiotherapy
OS: Overall survival
LCRT: Long-course radiation therapy
TNT: Total neoadjuvant therapy
PDL-1: Programmed death ligand- 1
PFS: Progression-free survival
TIL: Tumor infiltrating lymphocytes
PD-1: Programmed death- 1
SEER: Surveillance, Epidemiology, and End Results
R0 resection: Resection with negative margins
DFS: Disease-free survival
NCCN: National Comprehensive Cancer Network
QoL: Quality of life
AUBMC: American University of Beirut Medical Center
HDF: Hotel Dieu De France
KHCC: King Hussein Cancer Center
CRO: Contract research organization
ICH-GCP: International guidelines on Good Clinical Practice
SAE: Serious adverse event
IMP: Investigational medicinal product
FACT-C: Functional Assessment of Cancer Therapy in patients with colorectal cancer
D_n_: Day number n
IMRT: Intensity-modulated radiotherapy
MSI: Microsatellite instability
TRG: Tumor regression grading
CR: Complete response
Anti-CTLA4: Monoclonal antibody against cytotoxic T-lymphocyte–associated antigen 4
TAA: Tumor-associated antigens
APC: Antigen-presenting cell
CTL: Cytotoxic T-lymphocytes
IEC: Independent ethics committee
AE: Adverse event
eCRF: Electronic Case Report Form
DSMB: Data and Safety Monitoring Board
